# Association of HLA‐B22 serotype with SARS‐CoV‐2 susceptibility in Hong Kong Chinese patients

**DOI:** 10.1111/tan.14135

**Published:** 2020-12-01

**Authors:** Yuk‐Lin Yung, Chi‐Keung Cheng, Hoi‐Yun Chan, Jenny T. Xia, Kin‐Mang Lau, Raymond S. M. Wong, Alan K. L. Wu, Raymond W. Chu, Alice C. C. Wong, Eudora Y. D. Chow, Sze‐Fai Yip, Jennifer N. S. Leung, Cheuk‐Kwong Lee, Margaret H. L. Ng

**Affiliations:** ^1^ Blood Cancer Cytogenetics and Genomics Laboratory, Department of Anatomical and Cellular Pathology Prince of Wales Hospital, The Chinese University of Hong Kong Hong Kong China; ^2^ Department of Medicine and Therapeutics Prince of Wales Hospital, The Chinese University of Hong Kong Hong Kong China; ^3^ Sir Y. K. Pao Centre for Cancer Prince of Wales Hospital Hong Kong China; ^4^ Department of Clinical Pathology Pamela Youde Nethersole Eastern Hospital Hong Kong China; ^5^ Department of Pathology United Christian Hospital Hong Kong China; ^6^ Department of Medicine Tuen Mun Hospital Hong Kong China; ^7^ Hong Kong Red Cross Blood Transfusion Service Hong Kong China; ^8^ State Key Laboratory of Translational Oncology The Chinese University of Hong Kong Hong Kong China

**Keywords:** Chinese, coronavirus, COVID‐19, HLA, SARS‐CoV‐2

## Abstract

The coronavirus disease 2019 (COVID‐19) is a highly infectious disease caused by SARS‐CoV‐2. Since its first report in December 2019, COVID‐19 has evolved into a global pandemic causing massive healthcare and socioeconomic challenges. HLA system is critical in mediating anti‐viral immunity and recent studies have suggested preferential involvement of HLA‐B in COVID‐19 susceptibility. Here, by investigating the HLA‐B genotypes in 190 unrelated Chinese patients with confirmed COVID‐19, we identified a significant positive association between the B22 serotype and SARS‐CoV‐2 infection (*p* = 0.002, Bonferroni‐corrected *p* = 0.032). Notably, the B22 serotype has been consistently linked to susceptibility to other viral infections. These data not only shed new insights into SARS‐CoV‐2 pathogenesis and vaccine development but also guide better infection prevention/control.

COVID‐19 has been declared as a global pandemic by the World Health Organization (WHO) on 11 March 2020 and posed a major threat to public health worldwide. As of August 2020, the reported death toll in the world has surpassed 0.7 million among 20 million confirmed cases (https://covid19.who.int/). The disease is caused by a new and highly transmissible coronavirus named SARS‐CoV‐2, whose genomic sequence was 96% and 79.5% similar to that of bat SARS‐like coronavirus and the previous SARS‐CoV, respectively.^1^ The main symptoms of COVID‐19 are fever, cough and fatigue but there are also considerable numbers of infected cases who are asymptomatic, making the diagnosis and control of virus spread difficult. In addition, while the majority of the patients have mild symptoms, some severe cases can progress to acute respiratory distress syndrome or multiorgan dysfunction. While certain clinicopathological variables including older age, chronic diseases and lymphopenia are potentially linked to increased severity, other factors that affect SARS‐CoV‐2 susceptibility and the heterogeneity in clinical response remain largely unclear.

HLA plays a central role in the immune system by its involvement in the presentation of antigenic peptides to T‐cells to mediate anti‐viral immunity. The ubiquitously expressed HLA class I molecules are responsible for CD8 T‐cell response. On the other hand, class II molecules are mainly expressed on antigen presenting cells and mediate CD4 T‐cell response. As HLA alleles are highly polymorphic, individuals with different HLA genotypes may exhibit differential immune responses toward pathogen infection, thereby contributing to varied disease susceptibility and outcomes. In fact, previous studies from others and our group have revealed the association of HLA genotypes with SARS‐CoV infection susceptibility and severity.^2^, ^3^ Interestingly, a recent *in silico* analysis of the binding affinity between HLA class I molecules (including HLA‐A, ‐B and ‐C) and peptides derived from SARS‐CoV‐2 indicated that certain HLA‐B alleles might be particularly related to SARS‐CoV‐2 vulnerability.^4^ Accordingly, a recent report has also revealed a higher frequency of HLA‐B*15:27 in COVID‐19 patients than in the control population,^5^ further implying a differential role of HLA‐B in dictating SARS‐CoV‐2 susceptibility. In the present study, we sought to investigate the relationship of HLA‐B genotypes with susceptibility or resistance to SARS‐CoV‐2 infection and disease presentation in a cohort of 190 unrelated ethnic Chinese patients with confirmed COVID‐19 from Hong Kong.

The patient cohort included 96 males and 94 females who were admitted to six different local hospitals in Hong Kong from April to June 2020. First‐degree relatives among the patients had been excluded. The median age of the patients was 32 years (range 17‐93 years) and the diagnosis of COVID‐19 was made by detection of SARS‐CoV‐2 viral RNA in their deep throat saliva, nasopharyngeal swab or throat swab specimens by RT‐PCR according to the WHO guidelines. COVID‐19 disease severity was classified as mild/moderate, severe or critical as previously described.^6^ For control comparison, we obtained genotyping data of 3892 unrelated ethnic Chinese in the Hong Kong Chinese Cord Blood Registry (HKCCBR) from the Allele Frequency Net Database (AFND).^7^, ^8^ This study was approved by the Joint CUHK‐NTEC Clinical Research Ethics Committee and carried out following the Declaration of Helsinki.

Genomic DNA was extracted from peripheral blood with the Gentra Puregene Blood Kit (Qiagen) according to the manufacturer manual. HLA‐B genotypes were determined by Sanger sequencing‐based typing as previously described^9^ with the use of the SBTengine software (GenDx, Version 3.20.1) for allele assignment. Odds ratio (OR) with 95% confidence interval (CI) was obtained from a 2 × 2 contingency table. Statistical analysis was performed by Fisher's exact test with Bonferroni correction for multiple comparisons. Two‐sided *p* < 0.05 was considered statistically significant.

Thirty‐three different HLA‐B alleles were identified in the COVID‐19 patient cohort. Compared to the HKCCBR control, we observed higher frequencies of B*54:01, B*56:01 and B*56:04 in the patient group (*p* < 0.05) (Supporting Information, Table S1). Of note, all these three HLA‐B alleles belong to the same B22 serotype. Next, we grouped the alleles into serological subtypes (n = 15) for analysis. Consistently, the B22 serotype was found to be strongly associated with the COVID‐19 group (OR = 1.71, 95% CI = 1.23–2.38, *p* = 0.002) and the association remained statistically significant after Bonferroni correction (corrected *p* = 0.032) (Table 1). To validate these observations, we recruited 294 healthy blood donors (162 males, 132 females) who were ethnic Chinese and age‐matched with the COVID‐19 patient group from the Hong Kong Red Cross Blood Transfusion Service (HKRCBTS). The HLA‐B frequencies of the 15 serotypes in the recruited blood donors were found to be highly concordant with those in the HKCCBR (*r* = 0.987, *p* < 0.0001). Again, the B22 serotype showed higher frequencies in the COVID‐19 patient group than the HKRCBTS blood donors (OR = 1.66, 95% CI = 1.06–2.59, *p* = 0.027) though no corrected statistical significance was reached (Table 1). On the contrary, the B27 serotype was consistently found to be less frequent in the COVID‐19 patient group than the HKCCBR (*p* = 0.064) and HKRCBTS (*p* = 0.047) controls. The impacts of the B5 and B12 serotypes were inconsistently observed in the two control cohorts.

**TABLE 1:** The frequency of HLA‐B serotypes in the COVID‐19 patient group, the Hong Kong Chinese Cord Blood Registry (HKCCBR), and the Hong Kong Red Cross Blood Transfusion Service (HKRCBTS) blood donors

Serotype														
	COVID‐19 patients (n = 190)		HKCCBR (n = 3892)						HKRCBTS blood donors (n = 294)					
	Count	Frequency	Count*	Frequency	OR	95% CI	*p*‐value	Corrected *p*‐value	Count	Frequency	OR	95% CI	*p*‐value	Corrected *p*‐value
B5	15	3.95%	520	6.68%	0.57	0.34–0.97	0.033	ns	26	4.42%	/	/	0.870	/
B7	8	2.11%	123	1.58%	/	/	0.400	/	9	1.53%	/	/	0.618	/
B8	1	0.26%	23	0.30%	/	/	1.000	/	1	0.17%	/	/	1.000	/
B12	1	0.26%	143	1.84%	0.14	0.02–1.01	0.015	ns	5	0.85%	/	/	0.413	/
B13	38	10.00%	733	9.42%	/	/	0.719	/	59	10.03%	/	/	1.000	/
B15	69	18.16%	1217	15.63%	/	/	0.194	/	108	18.37%	/	/	1.000	/
B16	22	5.79%	556	7.14%	/	/	0.357	/	44	7.48%	/	/	0.361	/
B17	30	7.89%	673	8.65%	/	/	0.708	/	46	7.82%	/	/	1.000	/
B22	43	11.32%	540	6.94%	1.71	1.23–2.38	0.002	0.032	42	7.14%	1.66	1.06–2.59	0.027	ns
B27	4	1.05%	202	2.60%	/	/	0.064	/	18	3.06%	0.34	0.11–1.00	0.047	ns
B35	13	3.42%	259	3.33%	/	/	0.883	/	19	3.23%	/	/	0.856	/
B40	73	19.21%	1416	18.19%	/	/	0.634	/	105	17.86%	/	/	0.611	/
B46	56	14.74%	1090	14.00%	/	/	0.705	/	85	14.46%	/	/	0.926	/
B48	6	1.58%	147	1.89%	/	/	0.846	/	11	1.87%	/	/	0.807	/
B67	1	0.26%	14	0.18%	/	/	0.511	/	1	0.17%	/	/	1.000	/

Abbreviations: n, number of subjects; OR, Odds ratio; 95% CI, 95% confidence interval; corrected *p* value, *p* value after Bonferroni correction.

^a^ All the HLA‐B alleles belonging to the same serotype were counted.

Of the 181 COVID‐19 patients with follow‐up clinical data (Table S2), 21 cases (12%) had no symptoms at admission. Sixty patients (33%) had lymphopenia, which was noted in 40 cases on admission. The remaining 20 cases developed the abnormality 1 to 61 days after admission (median 3 days). Most of the symptomatic patients (150 of 160, 94%) had mild/moderate disease, whereas four and six cases had severe and critical illness, respectively. All the patients were alive at the last follow‐up. Interestingly, we observed that the putative SARS‐CoV‐2‐resistant B27 serotype tended to be over‐represented in asymptomatic patients (*p* = 0.068). No significant association of HLA‐B, both at allele and serotype levels, with lymphopenia and disease severity was found (Table 2).

**TABLE 2:** Relationship of HLA‐B serotypes with the presence of symptoms, lymphopenia, and disease severity in COVID‐19 patients

Serotype	Presence of symptoms					Lymphopenia					Disease severity*				
	Yes (n = 160)		No (n = 21)			Yes (n = 60)		No (n = 121)			Mild/moderate (n = 150)		Severe/critical (n = 10)		
	Count	Frequency	Count	Frequency	*p*‐value	Count	Frequency	Count	Frequency	*p*‐value	Count	Frequency	Count	Frequency	*p*‐value
B5	13	4.06%	2	4.76%	0.689	3	2.50%	12	4.96%	0.402	13	4.33%	0	0%	1.000
B7	8	2.50%	0	0%	0.604	4	3.33%	4	1.65%	0.448	8	2.67%	0	0%	1.000
B8	1	0.31%	0	0%	1.000	0	0%	1	0.41%	1.000	1	0.33%	0	0%	1.000
B12	0	0%	1	2.38%	0.116	0	0%	1	0.41%	1.000	0	0%	0	0%	1.000
B13	33	10.31%	4	9.52%	1.000	13	10.83%	24	9.92%	0.854	31	10.33%	2	10%	1.000
B15	62	19.38%	6	14.29%	0.531	19	15.83%	49	20.25%	0.391	58	19.33%	4	20%	1.000
B16	22	6.88%	0	0%	0.091	10	8.33%	12	4.96%	0.244	22	7.33%	0	0%	0.379
B17	25	7.81%	5	11.90%	0.370	9	7.50%	21	8.68%	0.840	24	8%	1	5%	1.000
B22	35	10.94%	4	9.52%	1.000	16	13.33%	23	9.50%	0.283	32	10.67%	3	15%	0.469
B27	2	0.63%	2	4.76%	0.068	1	0.83%	3	1.24%	1.000	2	0.67%	0	0%	1.000
B35	11	3.44%	1	2.38%	1.000	4	3.33%	8	3.31%	1.000	11	3.67%	0	0%	1.000
B40	58	18.13%	9	21.43%	0.672	22	18.33%	45	18.60%	1.000	52	17.33%	6	30%	0.224
B46	44	13.75%	7	16.67%	0.637	16	13.33%	35	14.46%	0.873	41	13.67%	3	15%	0.745
B48	5	1.56%	1	2.38%	0.526	3	2.50%	3	1.24%	0.403	4	1.33%	1	5%	0.277
B67	1	0.31%	0	0%	1.000	0	0%	1	0.41%	1.000	1	0.33%	0	0%	1.000

Abbreviation: n, number of subjects.

^a^ Disease severity was classified as mild/moderate (mild symptoms up to mild pneumonia), severe (dyspnea, hypoxia, or >50% lung involvement on imaging) or critical (respiratory failure, shock, or multi‐organ system dysfunction) according to the WHO guidelines.

To date, no specific treatment plan or vaccine is available for the highly infectious COVID‐19. Identification of high‐risk subjects susceptible to SARS‐CoV‐2 infection is of great importance in preventing virus spread, reducing public health burden and prioritizing preventive medicine. HLA class I molecules play a crucial role in directing anti‐viral immune responses and their genetic heterogeneity thus dictates clinical reactions and outcomes among individuals. Here, we showed that the HLA‐B22 serotype is a potential risk marker for SARS‐CoV‐2 infection. B22 is a broad antigen serotype including B54, B55 and B56 and has a frequency of about 7% in Asians and 2.6% in Europeans inferred from the AFND.^8^ This serotype has been implicated in poor immune response conferring susceptibility to several viral infectious diseases including hepatitis C virus (HCV) and human immunodeficiency virus (HIV).^10^, ^11^ Interestingly, a recent study by Barquera et al found that five B22 alleles (B*54:01, B*55:01, B*55:07, B*55:12 and B*56:01) were among the 94 weakest HLA‐B binders to SARS‐CoV‐2,^12^ further suggesting B22 as a susceptibility marker. On the other hand, our data seem to suggest a possible role of the HLA‐B27 serotype in modulating SARS‐CoV‐2 infection. B27 serotype is well‐known for its strong association with ankylosing spondylitis,^13^ but notably, this serotype has also been shown to mediate protection against HCV and HIV.^13^ The observation that the same HLA markers may be associated with susceptibility/resistance to all three different SARS‐CoV‐2, HIV and HCV may imply a common general immune mechanism operating against these viral infections. However, it can also implicate similarities in the cellular attacks in viral pathogenesis. Indeed, all the three viruses are RNA viruses. In particular, SARS‐CoV‐2 and HCV are positive‐sense RNA viruses that share striking sequence and structural homology in their RNA‐dependent RNA polymerase and protease, which are central components for viral replication.^14^, ^15^ Concordantly, hepatic manifestations have also been reported in COVID‐19 patients and are more prevalent in severe than in mild cases.^16^ On the other hand, lymphopenia is the clinical hallmark of HIV infection. This abnormality is also an important feature associated with increased COVID‐19 severity.^17^ Together, these observations suggest that perturbation of immune homeostasis also plays a significant role in the pathogenesis of this new coronavirus.

A recent study on 82 Chinese COVID‐19 patients from East China suggested HLA‐B*15:27 as a susceptibility marker.^5^ This allele has a frequency of 0.46% in the HKCCBR^7^ and was absent in our COVID‐19 patients and HKRCBTS blood donors, the majority of whom are southern Chinese. HLA‐B*46:01 was recently predicted by in silico studies to have the fewest binding peptides for SARS‐CoV‐2 and thus expected to confer disease susceptibility.^4^ We did not observe such associations here, possibly reflecting the notion that T‐cell‐mediated response is a highly complex and multiparameter process involving various factors not limited to HLA‐peptide interactions.^18^ Also, as most of our COVID‐19 patients had mild/moderate symptoms, this might have precluded the recognition of the effects of HLA polymorphisms on disease severity. It should be stated that the number of cases in our study is relatively limited, in particular those carrying the B27 serotype, and larger studies are warranted to confirm the findings. In addition, we did not study other HLA class I and II molecules here so their influences are unclear and await further investigations.

In conclusion, we have identified potential HLA markers that are related to the susceptibility and resistance to SARS‐CoV‐2. These findings will provide new insights into SARS‐CoV‐2 pathogenesis, the design of vaccination programs, and more effective infection control to reduce morbidity and mortality.

## CONFLICT OF INTEREST

The authors have declared no conflicting interests.

## AUTHOR CONTRIBUTIONS

Y.‐L.Y. and C.‐K.C. performed research, analyzed the data and wrote the manuscript. H.‐Y.C. and J.T.X. performed research and analyzed the data. K.‐M.L. analyzed the data and advised on revision of the manuscript. R.S.M.W., A.K.L.W., R.W.C., A.C.C.W., E.Y.D.C., S.‐F.Y., J.N.S.L. and C.‐K.L. recruited subjects and collected clinical data. M.H.L.N. designed and coordinated research and advised on revision of the manuscript. All authors read and approved the final manuscript.

## Supporting information


**Table S1** Comparison of HLA‐B allele frequency between the COVID‐19 patient and the Hong Kong Chinese Cord Blood Registry (HKCCBR) control groups.
**Table S2**. HLA‐B genotypes and clinical information of the 190 COVID‐19 patients.

## Data Availability

HLA‐B genotypes and clinical information of the COVID‐19 patients are disclosed in the supplementary information.
