# Supplementary material for: Association of HLA‐B22 serotype with SARS‐CoV‐2 susceptibility in Hong Kong Chinese patients
Source: HLA. 2020 Dec 1;97(2):127–32. doi: 10.1111/tan.14135 (PMC7898481; doi:10.1111/tan.14135)
Supplement: Supplementary file 1 — Table S1 Comparison of HLA‐B allele frequency between the COVID‐19 patient and the Hong Kong Chinese Cord Blood Registry (HKCCBR) control groups. Table S2. HLA‐B genotypes and clinical information of the 190 COVID‐19 patients. [file TAN-97-127-s001.docx]

**Supplemental Table 1.** Comparison of HLA-B allele frequency between the COVID-19 patient and the Hong Kong Chinese Cord Blood Registry (HKCCBR) control groups.

| **HLA Allele** | **HLA Serotype** | **COVID-19 patients (n=190)** | | **HKCCBR (n=3,892)** | | |
| --- | --- | --- | --- | --- | --- | --- |
|  |  | Allele count | Allele frequency | Allele count | Allele frequency | *P* value |
| B*51:01 | B5 | 9 | 2.37% | 335 | 4.30% | 0.067 |
| B*51:02 | B5 | 3 | 0.79% | 101 | 1.30% | 0.635 |
| B*52:01 | B5 | 3 | 0.79% | 74 | 0.95% | 1.000 |
| B*07:02 | B7 | 2 | 0.53% | 30 | 0.39% | 0.660 |
| B*07:05 | B7 | 6 | 1.58% | 93 | 1.19% | 0.467 |
| B*08:01 | B8 | 1 | 0.26% | 23 | 0.30% | 1.000 |
| B*44:02 | B12 | 1 | 0.26% | 17 | 0.22% | 0.576 |
| B*13:01 | B13 | 38 | 10.00% | 599 | 7.70% | 0.116 |
| B*15:01 | B15 | 16 | 4.21% | 218 | 2.80% | 0.114 |
| B*15:02 | B15 | 40 | 10.53% | 708 | 9.10% | 0.362 |
| B*15:11 | B15 | 4 | 1.05% | 44 | 0.57% | 0.284 |
| B*15:12 | B15 | 4 | 1.05% | 63 | 0.81% | 0.554 |
| B*15:18 | B15 | 2 | 0.53% | 38 | 0.49% | 0.710 |
| B*15:21 | B15 | 1 | 0.26% | 3 | 0.04% | 0.174 |
| B*15:25 | B15 | 1 | 0.26% | 86 | 1.10% | 0.192 |
| B*15:58 | B15 | 1 | 0.26% | 1 | 0.01% | 0.091 |
| B*38:01 | B16 | 1 | 0.26% | 33 | 0.42% | 1.000 |
| B*38:02 | B16 | 15 | 3.95% | 381 | 4.89% | 0.464 |
| B*39:01 | B16 | 6 | 1.58% | 132 | 1.70% | 1.000 |
| B*57:01 | B17 | 2 | 0.53% | 19 | 0.24% | 0.256 |
| B*58:01 | B17 | 28 | 7.37% | 654 | 8.40% | 0.568 |
| **B*54:01** | **B22** | **18** | **4.74%** | **202** | **2.60%** | **0.021** |
| B*55:02 | B22 | 17 | 4.47% | 257 | 3.30% | 0.240 |
| **B*56:01** | **B22** | **6** | **1.58%** | **50** | **0.64%** | **0.045** |
| **B*56:04** | **B22** | **2** | **0.53%** | **4** | **0.05%** | **0.029** |
| B*27:04 | B27 | 4 | 1.05% | 179 | 2.30% | 0.151 |
| B*35:01 | B35 | 11 | 2.89% | 148 | 1.90% | 0.179 |
| B*35:03 | B35 | 2 | 0.53% | 62 | 0.80% | 0.770 |
| B*40:01 | B40 | 65 | 17.11% | 1,214 | 15.60% | 0.427 |
| B*40:06 | B40 | 8 | 2.11% | 86 | 1.10% | 0.082 |
| B*46:01 | B46 | 56 | 14.74% | 1,090 | 14.00% | 0.705 |
| B*48:01 | B48 | 6 | 1.58% | 93 | 1.19% | 0.467 |
| B*67:01 | B67 | 1 | 0.26% | 14 | 0.18% | 0.511 |

n, number of subjects

**Supplemental Table 2.** HLA-B genotypes and clinical information of the 190 COVID-19 patients.

| **Case ID** | **Sex** | **Age** | **HLA-B allele 1** | **HLA-B allele 2** | **Lymphopenia** | **Number of days from admission to onset of lymphopenia** | **Symptomatic** | **Disease severity*** |
| --- | --- | --- | --- | --- | --- | --- | --- | --- |
| 1 | F | 32 | B*46:01 | B*58:01 | Yes | 0 | No | NA |
| 2 | M | 59 | B*46:01 | B*58:01 | No | NA | Yes | mild/moderate |
| 3 | M | 53 | B*40:01 | B*46:01 | No | NA | Yes | mild/moderate |
| 4 | M | 19 | B*46:01 | B*54:01 | No | NA | No | NA |
| 5 | M | 58 | B*15:01 | B*54:01 | No | NA | Yes | mild/moderate |
| 6 | M | 28 | B*35:01 | B*40:01 | No | NA | Yes | mild/moderate |
| 7 | M | 19 | B*15:02 | B*46:01 | No | NA | Yes | mild/moderate |
| 8 | M | 40 | B*15:12 | B*58:01 | No | NA | No | NA |
| 9 | F | 36 | B*35:01 | B*46:01 | Yes | 0 | Yes | mild/moderate |
| 10 | F | 27 | B*46:01 | B*67:01 | No | NA | Yes | mild/moderate |
| 11 | M | 49 | B*15:01 | B*58:01 | No | NA | Yes | mild/moderate |
| 12 | M | 65 | B*40:01 | B*40:06 | No | NA | No | NA |
| 13 | M | 18 | B*38:02 | B*46:01 | No | NA | Yes | mild/moderate |
| 14 | F | 71 | B*39:01 | B*58:01 | No | NA | Yes | mild/moderate |
| 15 | F | 19 | B*40:06 | B*54:01 | Yes | 0 | Yes | mild/moderate |
| 16 | M | 20 | B*27:04 | B*40:01 | No | NA | No | NA |
| 17 | M | 19 | B*27:04 | B*40:01 | No | NA | No | NA |
| 18 | M | 61 | B*15:02 | B*15:12 | No | NA | Yes | mild/moderate |
| 19 | M | 66 | B*15:11 | B*40:01 | Yes | 0 | Yes | mild/moderate |
| 20 | M | 51 | B*13:01 | B*54:01 | Yes | 0 | Yes | mild/moderate |
| 21 | M | 48 | B*15:01 | B*39:01 | No | NA | Yes | mild/moderate |
| 22 | F | 56 | B*38:02 | B*46:01 | Yes | 0 | Yes | mild/moderate |
| 23 | M | 25 | B*46:01 | B*46:01 | No | NA | Yes | mild/moderate |
| 24 | F | 37 | B*51:02 | B*54:01 | No | NA | Yes | mild/moderate |
| 25 | M | 27 | B*15:11 | B*58:01 | Yes | 0 | Yes | mild/moderate |
| 26 | F | 34 | B*15:02 | B*55:02 | No | NA | Yes | mild/moderate |
| 27 | F | 31 | B*13:01 | B*15:01 | No | NA | Yes | mild/moderate |
| 28 | M | 18 | B*40:01 | B*46:01 | No | NA | No | NA |
| 29 | F | 31 | B*13:01 | B*15:11 | No | NA | Yes | mild/moderate |
| 30 | M | 35 | B*15:21 | B*38:02 | No | NA | Yes | mild/moderate |
| 31 | M | 42 | B*46:01 | B*56:01 | Yes | 0 | No | NA |
| 32 | F | 26 | B*13:01 | B*46:01 | No | NA | Yes | mild/moderate |
| 33 | F | 49 | B*13:01 | B*38:02 | No | NA | Yes | mild/moderate |
| 34 | F | 24 | B*40:01 | B*54:01 | No | NA | Yes | mild/moderate |
| 35 | F | 32 | B*07:05 | B*39:01 | Yes | 0 | Yes | mild/moderate |
| 36 | M | 63 | B*07:02 | B*38:02 | Yes | 6 | Yes | mild/moderate |
| 37 | F | 63 | B*15:02 | B*40:01 | Yes | 0 | Yes | mild/moderate |
| 38 | M | 19 | B*15:02 | B*48:01 | No | NA | Yes | mild/moderate |
| 39 | F | 23 | B*15:02 | B*15:18 | No | NA | Yes | mild/moderate |
| 40 | F | 54 | B*13:01 | B*54:01 | Yes | 0 | Yes | mild/moderate |
| 41 | M | 25 | B*15:02 | B*52:01 | Yes | 0 | No | NA |
| 42 | M | 50 | B*38:02 | B*40:01 | Yes | 0 | Yes | mild/moderate |
| 43 | M | 29 | B*13:01 | B*40:01 | Yes | 0 | Yes | mild/moderate |
| 44 | F | 29 | B*13:01 | B*55:02 | No | NA | Yes | mild/moderate |
| 45 | F | 33 | B*40:01 | B*40:01 | No | NA | Yes | mild/moderate |
| 46 | M | 50 | B*46:01 | B*54:01 | Yes | 0 | Yes | mild/moderate |
| 47 | F | 72 | B*40:01 | B*58:01 | Yes | 0 | Yes | mild/moderate |
| 48 | M | 28 | B*13:01 | B*55:02 | Yes | 3 | Yes | critical |
| 49 | F | 70 | B*13:01 | B*55:02 | Yes | 0 | Yes | mild/moderate |
| 50 | M | 68 | B*15:02 | B*40:01 | Yes | 1 | Yes | severe |
| 51 | M | 20 | B*15:02 | B*40:01 | No | NA | Yes | mild/moderate |
| 52 | F | 20 | B*13:01 | B*46:01 | No | NA | Yes | mild/moderate |
| 53 | F | 18 | B*13:01 | B*15:02 | No | NA | Yes | mild/moderate |
| 54 | M | 39 | B*55:02 | B*58:01 | Yes | 0 | Yes | mild/moderate |
| 55 | F | 36 | B*13:01 | B*46:01 | No | NA | Yes | mild/moderate |
| 56 | M | 24 | B*15:01 | B*15:11 | No | NA | Yes | mild/moderate |
| 57 | F | 61 | B*07:02 | B*56:01 | No | NA | Yes | mild/moderate |
| 58 | F | 21 | B*13:01 | B*46:01 | Yes | 1 | Yes | mild/moderate |
| 59 | F | 33 | B*07:05 | B*35:01 | Yes | 5 | Yes | mild/moderate |
| 60 | M | 31 | B*15:01 | B*15:02 | Yes | 1 | Yes | mild/moderate |
| 61 | M | 28 | B*13:01 | B*40:01 | Yes | 6 | Yes | mild/moderate |
| 62 | F | 21 | B*15:02 | B*58:01 | No | NA | Yes | mild/moderate |
| 63 | M | 36 | B*13:01 | B*35:01 | Yes | 1 | Yes | mild/moderate |
| 64 | M | 65 | B*40:01 | B*48:01 | Yes | 0 | Yes | critical |
| 65 | M | 32 | B*13:01 | B*15:02 | Yes | 0 | Yes | mild/moderate |
| 66 | M | 20 | B*46:01 | B*51:01 | No | NA | Yes | mild/moderate |
| 67 | M | 62 | B*40:01 | B*46:01 | Yes | 0 | Yes | mild/moderate |
| 68 | F | 34 | B*40:01 | B*56:01 | No | NA | Yes | mild/moderate |
| 69 | M | 30 | B*15:01 | B*40:01 | No | NA | Yes | mild/moderate |
| 70 | M | 65 | B*15:01 | B*15:02 | Yes | 9 | Yes | mild/moderate |
| 71 | M | 25 | B*13:01 | B*38:02 | Yes | 3 | Yes | mild/moderate |
| 72 | F | 60 | B*15:02 | B*58:01 | No | NA | Yes | critical |
| 73 | M | 42 | B*15:02 | B*40:01 | No | NA | Yes | mild/moderate |
| 74 | M | 23 | B*13:01 | B*40:01 | No | NA | Yes | mild/moderate |
| 75 | M | 26 | B*15:02 | B*46:01 | Yes | 0 | Yes | mild/moderate |
| 76 | M | 24 | B*40:01 | B*46:01 | Yes | 3 | No | NA |
| 77 | M | 45 | B*38:02 | B*58:01 | No | NA | Yes | mild/moderate |
| 78 | F | 21 | B*48:01 | B*54:01 | Yes | 1 | Yes | mild/moderate |
| 79 | M | 24 | B*13:01 | B*58:01 | No | NA | Yes | mild/moderate |
| 80 | F | 19 | B*51:01 | B*58:01 | Yes | 0 | Yes | mild/moderate |
| 81 | F | 20 | B*15:58 | B*40:01 | No | NA | Yes | mild/moderate |
| 82 | M | 38 | B*15:01 | B*46:01 | Yes | 0 | Yes | mild/moderate |
| 83 | F | 61 | B*38:02 | B*38:02 | Yes | 2 | Yes | mild/moderate |
| 84 | M | 36 | B*15:02 | B*27:04 | No | NA | Yes | mild/moderate |
| 85 | F | 23 | B*15:02 | B*15:18 | Yes | 0 | Yes | mild/moderate |
| 86 | F | 19 | B*13:01 | B*13:01 | No | NA | Yes | mild/moderate |
| 87 | M | 20 | B*46:01 | B*57:01 | Yes | 17 | Yes | mild/moderate |
| 88 | F | 27 | B*38:02 | B*58:01 | Yes | 6 | Yes | mild/moderate |
| 89 | F | 21 | B*35:01 | B*46:01 | No | NA | Yes | mild/moderate |
| 90 | F | 36 | B*15:02 | B*46:01 | No | NA | Yes | mild/moderate |
| 91 | M | 67 | B*46:01 | B*56:01 | No | NA | Yes | severe |
| 92 | M | 59 | B*27:04 | B*40:01 | Yes | 0 | Yes | mild/moderate |
| 93 | M | 62 | B*07:05 | B*46:01 | Yes | 0 | Yes | mild/moderate |
| 94 | M | 21 | B*15:02 | B*58:01 | No | NA | Yes | mild/moderate |
| 95 | M | 29 | B*13:01 | B*46:01 | No | NA | Yes | mild/moderate |
| 96 | M | 33 | B*40:01 | B*58:01 | No | NA | Yes | mild/moderate |
| 97 | M | 24 | B*40:01 | B*56:01 | No | NA | Yes | mild/moderate |
| 98 | F | 57 | B*15:02 | B*40:01 | No | NA | Yes | mild/moderate |
| 99 | F | 57 | B*40:06 | B*55:02 | No | NA | Yes | mild/moderate |
| 100 | M | 28 | B*35:01 | B*55:02 | Yes | 0 | Yes | mild/moderate |
| 101 | M | 23 | B*40:01 | B*55:02 | No | NA | Yes | mild/moderate |
| 102 | F | 22 | B*15:02 | B*40:06 | No | NA | Yes | mild/moderate |
| 103 | F | 20 | B*15:01 | B*15:02 | No | NA | Yes | mild/moderate |
| 104 | F | 27 | B*13:01 | B*58:01 | No | NA | No | NA |
| 105 | M | 20 | B*13:01 | B*55:02 | No | NA | No | NA |
| 106 | F | 18 | B*15:02 | B*58:01 | No | NA | Yes | mild/moderate |
| 107 | F | 26 | B*15:02 | B*51:01 | No | NA | Yes | mild/moderate |
| 108 | F | 19 | B*46:01 | B*46:01 | No | NA | Yes | mild/moderate |
| 109 | F | 19 | B*40:01 | B*46:01 | No | NA | Yes | mild/moderate |
| 110 | M | 20 | B*46:01 | B*48:01 | Yes | 0 | Yes | mild/moderate |
| 111 | M | 23 | B*15:02 | B*40:01 | No | NA | Yes | mild/moderate |
| 112 | M | 58 | B*15:02 | B*51:01 | No | NA | Yes | mild/moderate |
| 113 | F | 62 | B*15:01 | B*54:01 | No | NA | Yes | mild/moderate |
| 114 | F | 23 | B*15:02 | B*46:01 | No | NA | Yes | mild/moderate |
| 115 | F | 27 | B*38:01 | B*40:01 | No | NA | Yes | mild/moderate |
| 116 | M | 19 | B*40:01 | B*58:01 | No | NA | Yes | mild/moderate |
| 117 | F | 61 | B*38:02 | B*40:01 | No | NA | Yes | mild/moderate |
| 118 | F | 19 | B*40:01 | B*46:01 | No | NA | Yes | mild/moderate |
| 119 | F | 29 | B*35:03 | B*51:01 | No | NA | Yes | mild/moderate |
| 120 | M | 60 | B*40:01 | B*40:06 | No | NA | Yes | mild/moderate |
| 121 | F | 35 | B*15:12 | B*46:01 | No | NA | Yes | mild/moderate |
| 122 | M | 26 | B*39:01 | B*51:02 | No | NA | Yes | mild/moderate |
| 123 | M | 18 | B*40:01 | B*46:01 | No | NA | Yes | mild/moderate |
| 124 | M | 21 | B*51:01 | B*55:02 | No | NA | Yes | mild/moderate |
| 125 | M | 56 | B*40:01 | B*51:01 | Yes | 0 | Yes | mild/moderate |
| 126 | F | 61 | B*15:02 | B*55:02 | No | NA | Yes | mild/moderate |
| 127 | F | 31 | B*13:01 | B*46:01 | Yes | 0 | Yes | mild/moderate |
| 128 | F | 21 | B*13:01 | B*35:01 | No | NA | No | NA |
| 129 | F | 58 | B*15:02 | B*38:02 | No | NA | Yes | mild/moderate |
| 130 | M | 58 | B*13:01 | B*46:01 | No | NA | Yes | mild/moderate |
| 131 | M | 19 | B*15:02 | B*58:01 | No | NA | No | NA |
| 132 | M | 18 | B*51:02 | B*55:02 | No | NA | No | NA |
| 133 | M | 59 | B*15:02 | B*40:06 | Yes | 0 | Yes | critical |
| 134 | F | 59 | B*15:02 | B*40:06 | No | NA | No | NA |
| 135 | M | 40 | B*13:01 | B*46:01 | No | NA | Yes | mild/moderate |
| 136 | M | 30 | B*40:01 | B*40:01 | No | NA | Yes | mild/moderate |
| 137 | F | 37 | B*52:01 | B*57:01 | No | NA | Yes | mild/moderate |
| 138 | F | 20 | B*15:02 | B*58:01 | No | NA | Yes | mild/moderate |
| 139 | F | 25 | B*40:01 | B*46:01 | No | NA | No | NA |
| 140 | F | 20 | B*08:01 | B*46:01 | No | NA | Yes | mild/moderate |
| 141 | F | 57 | B*40:01 | B*46:01 | No | NA | Yes | mild/moderate |
| 142 | F | 26 | B*40:01 | B*52:01 | No | NA | Yes | mild/moderate |
| 143 | M | 79 | B*40:01 | B*55:02 | No | NA | Yes | mild/moderate |
| 144 | F | 75 | B*15:02 | B*56:04 | Yes | 0 | Yes | mild/moderate |
| 145 | M | 22 | B*39:01 | B*58:01 | No | NA | Yes | mild/moderate |
| 146 | M | 38 | B*46:01 | B*58:01 | No | NA | Yes | mild/moderate |
| 147 | M | 20 | B*15:01 | B*54:01 | No | NA | Yes | mild/moderate |
| 148 | F | 63 | B*07:05 | B*40:01 | No | NA | Yes | mild/moderate |
| 149 | M | 22 | B*15:01 | B*44:02 | No | NA | No | NA |
| 150 | M | 20 | B*15:25 | B*48:01 | No | NA | No | NA |
| 151 | M | 20 | B*51:01 | B*54:01 | No | NA | Yes | mild/moderate |
| 152 | M | 56 | B*58:01 | B*58:01 | Yes | 0 | Yes | mild/moderate |
| 153 | F | 19 | B*15:02 | B*55:02 | No | NA | Yes | mild/moderate |
| 154 | F | 61 | B*07:05 | B*35:01 | No | NA | Yes | mild/moderate |
| 155 | F | 25 | B*13:01 | B*13:01 | No | NA | Yes | mild/moderate |
| 156 | M | 20 | B*48:01 | B*54:01 | No | NA | Yes | mild/moderate |
| 157 | F | 20 | B*13:01 | B*35:01 | No | NA | Yes | mild/moderate |
| 158 | F | 48 | B*15:01 | B*54:01 | No | NA | Yes | mild/moderate |
| 159 | F | 26 | B*07:05 | B*13:01 | No | NA | Yes | mild/moderate |
| 160 | F | 22 | B*46:01 | B*58:01 | No | NA | No | NA |
| 161 | F | 54 | B*40:01 | B*46:01 | No | NA | Yes | mild/moderate |
| 162 | M | 64 | B*40:01 | B*40:06 | Yes | 0 | Yes | mild/moderate |
| 163 | M | 24 | B*40:01 | B*58:01 | No | NA | Yes | mild/moderate |
| 164 | M | 79 | B*40:01 | B*46:01 | No | NA | Yes | severe |
| 165 | F | 26 | B*39:01 | B*55:02 | Yes | 1 | Yes | mild/moderate |
| 166 | F | 21 | B*46:01 | B*51:01 | No | NA | Yes | mild/moderate |
| 167 | F | 21 | B*13:01 | B*35:01 | No | NA | Yes | mild/moderate |
| 168 | M | 93 | B*40:01 | B*40:01 | Yes | 1 | Yes | mild/moderate |
| 169 | F | 35 | B*15:01 | B*38:02 | No | NA | Yes | mild/moderate |
| 170 | F | 59 | B*38:02 | B*55:02 | Yes | 1 | Yes | mild/moderate |
| 171 | F | 62 | B*40:01 | B*55:02 | Yes | 1 | Yes | mild/moderate |
| 172 | F | 18 | B*40:01 | B*58:01 | No | NA | Yes | mild/moderate |
| 173 | F | 52 | B*13:01 | B*40:01 | No | NA | Yes | mild/moderate |
| 174 | M | 37 | B*15:12 | B*54:01 | Yes | 0 | Yes | mild/moderate |
| 175 | F | 45 | B*13:01 | B*40:01 | Yes | 0 | No | NA |
| 176 | M | 57 | B*13:01 | B*56:01 | Yes | 0 | Yes | severe |
| 177 | M | 17 | B*35:03 | B*40:01 | No | NA | Yes | mild/moderate |
| 178 | F | 87 | B*15:02 | B*46:01 | No | NA | Yes | mild/moderate |
| 179 | M | 69 | B*40:01 | B*40:01 | Yes | 0 | Yes | critical |
| 180 | F | 75 | B*15:02 | B*46:01 | Yes | 0 | Yes | critical |
| 181 | F | 91 | B*15:02 | B*46:01 | Yes | 61 | Yes | mild/moderate |
| 182 | F | 34 | B*15:01 | B*35:01 | / | / | / | / |
| 183 | M | 56 | B*40:01 | B*40:01 | / | / | / | / |
| 184 | F | 57 | B*40:01 | B*46:01 | / | / | / | / |
| 185 | F | 68 | B*40:01 | B*54:01 | / | / | / | / |
| 186 | F | 56 | B*46:01 | B*46:01 | / | / | / | / |
| 187 | F | 78 | B*46:01 | B*54:01 | / | / | / | / |
| 188 | M | 72 | B*13:01 | B*54:01 | / | / | / | / |
| 189 | F | 58 | B*40:01 | B*40:01 | / | / | / | / |
| 190 | M | 33 | B*46:01 | B*56:04 | / | / | / | / |

Clinical information for case 182-190 are not available; NA, not applicable
